# Impact of Stabilization Splint Therapy on Orthodontic Diagnosis in Patients with Signs and Symptoms of Temporomandibular Disorder

**DOI:** 10.3390/biomedicines12102251

**Published:** 2024-10-03

**Authors:** Kenan Demirovic, Vildana Dzemidzic, Enita Nakas

**Affiliations:** 1 Private Practice for Orthodontics and Dentofacial Orthopedics, 71000 Sarajevo, Bosnia and Herzegovina; 2 Department of Orthodontics, School of Dental Medicine, University of Sarajevo, 71000 Sarajevo, Bosnia and Herzegovina; vdzemidzic@hotmail.com (V.D.); enitta@gmail.com (E.N.)

**Keywords:** centric relation, maximum intercuspation, lateral cephalogram, stabilization splint

## Abstract

Background/Objectives: The relation between the orthopedic temporomandibular joint (TMJ) instability and temporomandibular disorder (TMD) most commonly remain unrecognized by orthodontists. In this study we aimed to evaluate the dentofacial characteristics and temporomandibular disorder symptomatology of patients with orthopedic instability before and after deprogramming with a stabilization splint. Methods: Sixty patients with the signs and symptoms of TMD were assessed using the Research Diagnostic Criteria for Temporomandibular Disorders (RDC/TMD) and underwent stabilization splint therapy to place the condyles in a more stable musculoskeletal position. The extent of condylar displacement was evaluated using the condylar position indicator (CPI). Sixteen angular and linear hard tissue landmarks were traced and compared from lateral cephalograms taken in the maximum intercuspation (MI) position before, and in the centric relation (CR) position after, the splint therapy. Results: Following the splint therapy, the signs and symptoms of TMD were significantly reduced or completely eliminated in more than 90% of patients. Compared with the values registered before the splint therapy, a significant reduction in the mean values of condylar displacement was observed on both sides of the vertical (*p* < 0.001), horizontal (*p* < 0.05), and transverse (*p* < 0.001) planes of space after the splint therapy. A comparison of pre- and post-splint lateral cephalograms revealed that, following the splint therapy, the mandible moved more posteriorly and rotated in a more clockwise direction. Conclusions: In patients with orthopedic instability and the signs and symptoms of TMD, muscle deprogramming with a stabilization splint therapy is highly recommended to improve the health of the temporomandibular joint and masticatory structures and contribute to a more correct orthodontic diagnosis.

## 1. Introduction

Currently, it is widely accepted that temporomandibular disorders have a multifactorial etiology and different modalities of treatment [1,2]. Occlusal factors related to orthopedic instability, depicted by an increased centric relation (CR)–maximum intercuspation (MI) discrepancy at the dental and condylar levels, were classified as one of the most important factors involved in the etiology of temporomandibular and masticatory muscle disorders [3]. The position of the CR, the most orthopedically stable musculoskeletal position of the condyles in the fossa, is considered a highly reproducible reference point for the final post-orthodontic and post-prosthodontic reestablishing of the maxillo–mandibular relationship [4,5]. Orthopedically unstable patients are reported to develop a certain number of signs and symptoms that are related to temporomandibular joint or masticatory muscle disorders [6,7,8]. The treatment of orthodontic patients with signs and symptoms of TMD caused by an unstable musculoskeletal position represents a distinct, laborious, and time-consuming challenge for every orthodontist [9,10,11]. Based on his own and on earlier findings, Okeson emphasized the importance of orthopedic stability. “Establishing an orthopedically stable relationship between the occlusal position of the teeth and the joint position is important for a proper masticatory function throughout the patient’s lifetime. Although in most situations orthodontic therapy neither causes nor prevents TMD, the orthodontist is in an excellent position to provide orthopedic stability in the masticatory structures. Treatment goals directed toward establishing orthopedic stability in the masticatory structures should be a routine part of all orthodontic therapy. Achieving these goals will most likely reduce the patient’s risk factors for developing TMD” [12]. This implies that in patients with orthopedic instability, a stable position of the condyles in the fossa that considers the coincident positions of the CR and MI should be the primary goal of orthodontic treatment from the perspective of condylar and masticatory structure health [13,14]. Mounting diagnostic casts on an articulator and using a condylar position indicator (CPI) are recommended to evaluate the differences between the CR and MI at the condylar level and find the occlusal interferences responsible for those discrepancies [6,13,14]. Moreover, the measurement of condylar displacement using CPI instrumentation represents a very convenient way to evaluate the position of the condylar processes in three dimensions, as cases of transverse plane condylar displacement cannot be detected by means of clinical examination, an analysis of hand-articulated casts, or the use of 2D radiography. Studies comparing the extent of condylar displacement between the symptomatic and asymptomatic populations determined that condylar displacement was significantly greater in symptomatic individuals [6,15,16,17]. To assume its positioning in the MI mandible must avoid occlusal interferences and adopt a deviated pattern of closure, which results in condylar distraction followed by orthopedic instability and defective programming of the masticatory muscles. Orthopedic stability is most efficaciously achieved via stabilization splint therapy which, by deprogramming the muscle engrams, eliminates the patient’s neuromuscular response to a deviated closure pattern dictated by the habitual position of MI and thus induces muscle relaxation, reduces pain in muscles and TMJ structures, and has a positive effect on intracapsular disorders [8,18,19,20].

Establishing an orthopedically stable musculoskeletal position might induce substantial changes in the interarch relationship and thus alter the dentofacial characteristics of the patient, directly interfering with a correct orthodontic diagnosis [21,22]. Recent studies have confirmed that the stable musculoskeletal position provided by stabilization splint therapy represents a major objective when making the correct orthodontic diagnosis and evaluating the overall condylar health of orthodontic patients with TMD [23,24,25]. Even asymptomatic individuals with an increased CR–MI discrepancy need neuromuscular deprogramming to obtain a more accurate measurement of condylar displacement and to avoid inaccurate orthodontic diagnosis and treatment planning [26,27,28].

The first hypothesis of this study was that establishing orthopedic stability with a stabilization splint can have an impact on the orthodontic diagnosis after the condyles are placed in a stable musculoskeletal position. The second hypothesis was that there is a correlation between the signs and symptoms of TMD and the increased CR–MI differences measured at the condyle level using a CPI. The null hypothesis of this study was that there are no changes in the position of the condyle following stabilization splint therapy in comparison to the natural MI position.

The objectives of the study were as follows:

- To compare the mean values of sixteen variables from lateral cephalograms analyzed before and after establishing an orthopedically stable musculoskeletal position with stabilization splint therapy;
- To statistically compare the prevalence of the anamnestic and clinical findings of TMDs evaluated before and after establishing orthopedic stability in the masticatory structures with stabilization splint therapy;
- To evaluate the differences between the CR and MI at the level of the condyles using a CPI;
- To compare the prevalence of TMDs, condylar displacement, and cephalometric measurements among males and females.

## 2. Materials and Methods

### 2.1. Subjects

The sample for this study consisted of sixty patients, thirty females and thirty males (aged 18 to 30 years), randomly selected from patients who were referred to the orthodontic clinic. All subjects were diagnosed with an orthopedically unstable musculoskeletal position of the condyles in the fossa, presented with signs and symptoms of TMD, and were eligible for orthodontic therapy. Orthopedically unstable musculoskeletal position was determined at the occlusal and at the condylar levels. The bilateral mandibular guiding technique was used to position the condyles in their most anterosuperior position in the fossa via passive jaw manipulation to the first tooth contact [4]. At the dental level, the horizontal and vertical distances between the mandibular and maxillary incisors were measured in the positions of MI and CR using a Pittsburg digital caliper (Harbor Freight Tools, Calabasas, CA, USA). In each subject, the increased CR-MI discrepancy determined at the occlusal level was additionally confirmed at the level of the condyles using a condylar position indicator articulator (Panadent Corp, Grand Terrace, CA, USA). The cut-off value for the magnitude of CR-MI discrepancy at the occlusal level was 1.5 mm, wherein the cut-off values of >1 mm displacement for the vertical and horizontal planes and >0.5 mm for the transverse plane were considered significant at the condylar level. Out of the sixty patients included in the study, thirty-three (55%) subjects self-reported facial pain either in the temporomandibular joint and/or masticatory structures, while in twenty-seven (45%) subjects, pain and/or tenderness was diagnosed using the RDC/TMD protocol. The investigation was performed in the Demirović private practice for orthodontics and dentofacial orthopedics. The inclusion criteria were no history of orthodontic treatment, prosthetic treatment, orthognathic surgery, neurosurgery, TMJ treatment, head or jaw trauma, degenerative arthritis, muscle contracture, myositis, pain of odontogenic origin, or periodontal disease, and major irregularities related to condylar, coronoid and styloid processes. Patients with rheumatoid arthritis or other systemic diseases were excluded from the study. With regard to multifactorial etiology and the uniqueness of the TMJ structures, the inclusion of an equal number of females and males in the study was of particular significance. Ethical approval of the research protocol was provided by the Ethics Committee, School of Dental Medicine, University of Sarajevo.

### 2.2. Assessment of the Signs and Symptoms of TMD

A clinical assessment for TMD was performed according to the Research Diagnostic Criteria for Temporomandibular Disorders (RDC/TMD) before and after therapy with a stabilization splint. The RDC/TMD protocol comprises two axes: Axis I is used to assess the clinical signs and symptoms based on standardized diagnostic criteria, and Axis II is used to evaluate psychosocial distress and pain-related disability [29]. To perform an evaluation of clinical signs and subjective symptoms of TMD, the RDC/TMD instrument (Axis I) was used. All subjects filled out a questionnaire related to the subjective evaluation of facial pain, muscle and joint pain, headache, and parafunctional activities.

After the completion of the questionnaire, each patient underwent a clinical examination of the muscles and joints. The physical examination focused on the diagnosis of muscle and joint pain, the presence of joint noises on opening and closing, and the protrusion and retrusion and lateral excursion movements of the mandible. Measurements of mouth opening and the right and left excursion and protrusion movements of the mandible were performed using maximum unassisted extension during physical examination. Prior to palpation, finger pressure was calibrated to 0.5 or 1.0 kg depending on the area examined (temporomandibular joint structures, masticatory muscles) using an FPX 25 digital algometer (Wagner instruments, Greenwich, CT, USA).

A clinical assessment of all patients was conducted by the same trained examiner, an expert in the clinical evaluation of the signs and symptoms of TMD. In the study, an evaluation of psychosocial distress and pain-related disability according to Axis II was not performed because the operator lacked professional knowledge of the psychological and psychosocial assessment of study participants [30].

### 2.3. Assessment of Condylar Displacement before and after the Splint Therapy

**Figure 1:**
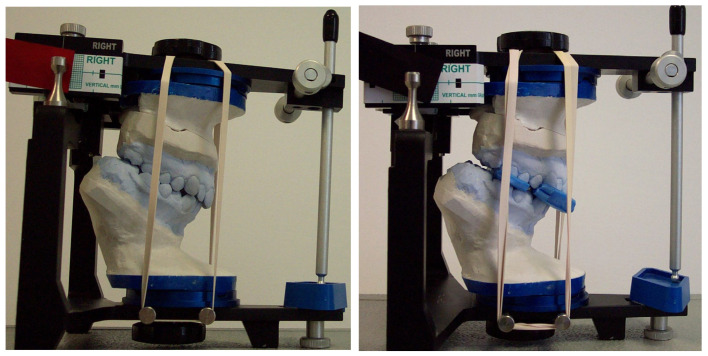
Diagnostic casts placed in the condylar position indicator (CPI) according to the initial MI and CR bite registration record to evaluate the CR–MI discrepancy before occlusal splint therapy.

To modify a muscle engram and assist in the recording of the mandible in the CR position, a leaf gauge (Panadent Corp, Grand Terrace, CA, USA) was placed between the anterior teeth to ensure an occlusal clearance between the posterior teeth. In all subjects, CR bite registrations were taken using a two-part (anterior and posterior) blue wax (Delar Corp, Lake Oswego, OR, USA) according to Roth’s power-centric technique. The anterior section of the wax was softened and folded in four layers over the incisor teeth region (canine–canine). The patients were asked to close gently within the arc of mandibular closure until approximately 2 mm of posterior disocclusion was obtained. To avoid distortion, the wax was cooled with air and placed in a glass with cold water to harden. The posterior section of the wax was folded into two layers and trimmed to fit over the buccal surfaces of the posterior teeth. With both wax sections in the mouth, the mandible was slowly guided by the chin-point into CR until the lower anterior teeth reached the previous indentations in the anterior section of the wax. For bite registration in the MI position, a single layer of hard pink wax (Beauty Pink Wax X Hard, Integra Miltex, York, PA, USA) was used. Diagnostic casts of all participants were mounted on a semi-adjustable articulator (Panadent Corp, Grand Terrace, CA, USA) using an estimated face-bow and CR bite registration. To obtain a high degree of accuracy and verification when mounting the articulator, a split cast method was used. The mounted models were placed in a condylar position indicator articulator to measure the extent of condylar discrepancy between the MI and CR positions using an initial MI and CR bite registration record (Figure 1).

**Figure 2:**
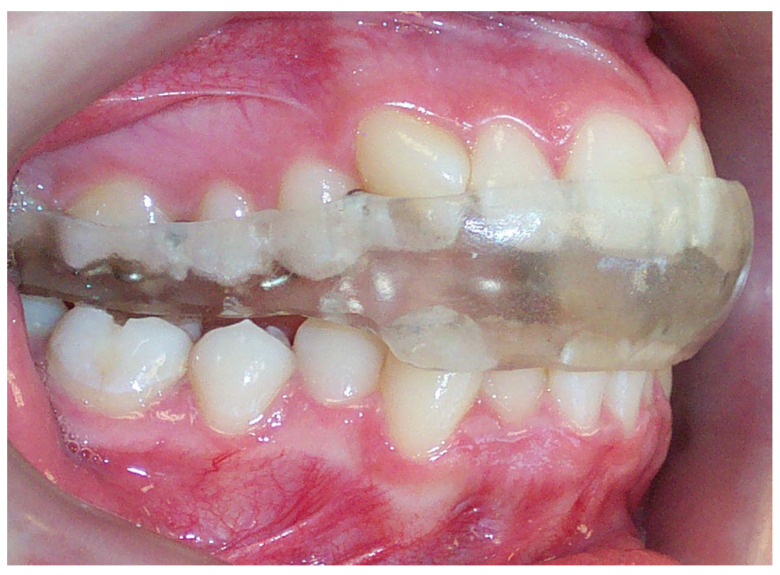
Stabilization splint.

For the purpose of achieving an orthopedically stable musculoskeletal position, flat plane occlusal stabilization splints were constructed according to the principles of a mutually protected occlusal scheme on models mounted in the CR position. Maxillary stabilization splints were made of heat-cured acrylic material (Interacryl Hot, Interdent, Opekarniska, Slovenia) using an indirect technique with Zetalabor condensation silicone (Zhermack, Badia Polesine, Italy). Stabilization splints were designed to provide uniform contact points of equal intensity on the anterior and posterior mandibular teeth. All participants used the acrylic stabilization splint for 24 h every day for approximately 6 months until a stable musculoskeletal position was achieved for the condyles in the CR (Figure 2).

At each visit, splint adjustments were carefully performed to provide even contact with the opposing teeth throughout the surface of the stabilization splint. CR records were taken every 15 days during the third and fourth month of splint wear and at one-week intervals during the fifth and sixth month of splint therapy in order to monitor condylar seating in an orthopedically stable position in the fossa. The indicator of a stable musculoskeletal position of the condyles in the fossa was three successive CR records with identical values measured at the condyle level using the CPI, unchanged uniform tooth contact throughout the dental arch during the last month of splint wear, without the need for further splint grinding, easy manipulation of the mandible, and a stable mandibular position. After orthopedic stability was achieved through the splint therapy, newly obtained dental casts were mounted on a semi-adjustable articulator according to the new CR bite registration. Subsequent to this, dental casts were placed in the CPI articulator, where the final MI and CR bite registrations were used to measure the condylar discrepancy between the CR and MI positions. The linear displacement of the condylar processes in a given axis was measured using graph paper and a magnifying glass with a 0.1 mm measuring lines. All procedures were performed by the same trained operator except for the articulator mountings, which were carried out by a laboratory technician. Following the stabilization splint therapy, all patients were diagnosed using cephalometric head films and underwent orthodontic therapy with fixed orthodontic appliances.

### 2.4. Radiologic Evaluation

Orthopantomograms (OPG) and lateral cephalograms (LCG) were obtained using Orthophos SL (Dentsply Sirona, Bensheim, Germany), a panoramic and cephalometric imaging system. OPG images were evaluated for major condylar irregularities, mandibular coronoid process hypertrophy, Eagle syndrome, and jaw tumors and cysts. All radiographic procedures performed by the radiology technician were in accordance with the principles of the radiographic technique used in the study and were the same for each patient. All radiographic images were taken at the School of Dental Medicine, University of Sarajevo. Lateral cephalograms of all subjects were taken in the natural head position (NHP). An initial lateral cephalogram (T^0^) was taken in the MI position before the splint therapy, and the final lateral cephalogram (T^1^) was taken in the CR position after the orthopedically stable musculoskeletal position was established with a stabilization splint. In order to take the final lateral cephalograms (T^1^) in the most accurate position, wax bites were constructed in the position of centric occlusion on dental casts mounted on a semi-adjustable articulator according to the new CR bite registration, obtained after orthopedic stability was achieved with splint therapy. Sixteen cephalometric variables related to the maxillo–mandibular relationship, vertical skeletal relationship, size of the mandible, and dental relationship were evaluated on lateral cephalograms taken in the MI position before the splint therapy and on lateral cephalograms taken in the CR position after the splint therapy. Digital tracings and superimpositions of the pre-splint and post-splint lateral cephalograms were performed with Dolphin Imaging software, version 11.8 (Dolphin Imaging and Management, Chatsworth, CA, USA) [31].

### 2.5. Statistical Analysis

The data are expressed as the mean ± standard deviation or number of cases and their percentage, depending on the type of data. Cronbach’s alpha reliability test was used to evaluate the reliability of the sample size.

To compare the tested signs and symptoms of TMD before and after the splint therapy, Chi-square and Fisher’s exact test were used, while the differences in dentofacial characteristics between the T0 and T1 and differences in the condylar displacements were evaluated using the Wilcoxon signed-rank test. Spearman’s correlation test was used to evaluate the relationship between the CR–MI differences and the signs and symptoms of TMD. The level of significance was set at 95% (*p* < 0.05). The data were compared using the statistical package IBM Statistics SPSS v 23.0 (IBM Corp., Armonk, NY, USA).

## 3. Results

A statistically significant difference was observed between the results for the anamnestic and diagnostic criteria of the RDC/TMD protocol evaluated before and after the splint therapy. Out of 60 patients, 33 (55%) reported recurrent (75.8%) or persistent (24.2%) facial pain before the start of the splint therapy. After the splint therapy, no patient reported persistent pain, recurrent pain was reported by one (3.0%), and one instance of pain was reported by two (6.1%) patients. RDC/TMD patients answered six questions on the history questionnaire regarding facial pain that were evaluated on a 0 to 10 numeric rating scale (NRS). When comparing the results obtained before and after the splint therapy, the level of facial pain reported by patients was significantly reduced after the splint therapy (Table 1).

**Figure 3:**
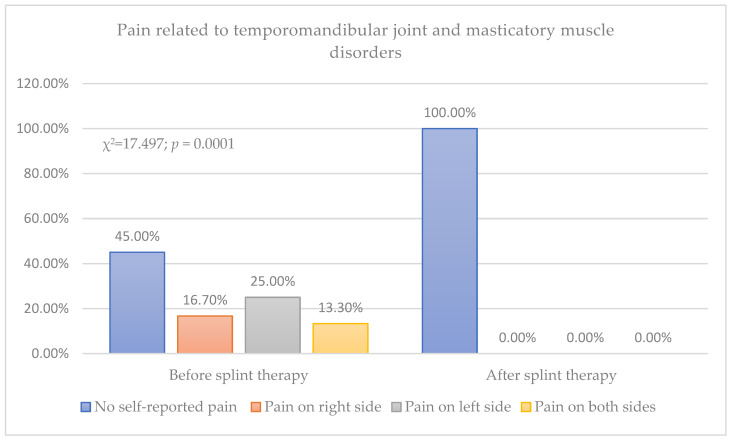
Presence of pain related to temporomandibular joint and masticatory muscle disorders.

Before the splint therapy, pain related to temporomandibular joint and masticatory muscle disorders was reported by 55% of patients, pain on the right side by 16.7%, pain on the left side by 25%, and pain on both sides by 13.3%, while after the splint therapy, none of the patients reported pain in these structures (*p* < 0.001) (Figure 3).

**Figure 4:**
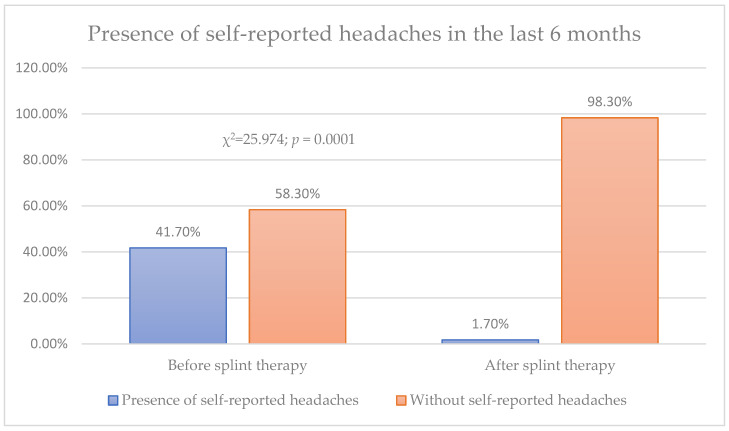
Presence of self-reported headaches in the six months prior to therapy.

The self-reported headache frequency was 41.7% before the splint therapy, and after the splint therapy, only 1.7% of patients reported headaches (Figure 4).

The self-reported prevalence of jaw clicking before the splint therapy was 70%, and after the splint therapy, it was reduced to 8.3% (*p* < 0.001). Before the splint therapy, 40% of participants reported a locked jaw, while after the therapy, none of the participants reported jaw locking (*p* < 0.001). Data regarding the other symptoms of TMD related to jaw dysfunction are presented in Table 2.

According to the physical findings, after the splint therapy, a statistically significant reduction in muscular and temporomandibular joint pain was achieved for mouth-opening and lateral excursion (*p* < 0.001). Sensitivity to palpation in the masticatory muscles and TMJ structures was recorded and graded as none, mild, moderate, or severe pain. Pain that was registered in the right and left masseter muscles (superficial, middle, and deep portions), temporalis (anterior, middle, posterior), posterior mandibular region (stylohyoid/posterior digastric region), or submandibular region (medial pterygoid/suprahyoid/anterior digastric region) before the splint therapy was significantly reduced or completely disappeared after the splint therapy (*p* < 0.05 and *p* < 0.001). Before the splint therapy, tenderness was present in 96.7% of patients on the left lateral pole and in 90% of patients on the right lateral pole, while after the therapy this was reduced to 8.3% on the left and 6.7% on the right lateral pole (*p* < 0.001). When comparing the results obtained before and after the splint therapy, a highly significant reduction in pain was observed in the lateral pterygoid area, tendon of temporalis, and posterior attachment after the splint therapy (*p* < 0.001).

When compared before and after, the mean CPI values of condylar displacement were significantly reduced on both sides of the vertical (*p* < 0.001), horizontal (*p* < 0.05), and transverse planes of space after the splint therapy (*p* < 0.001). Alongside these findings, a significant reduction was found in both the male and female groups in the mean CPI values of condylar displacements on the right and left sides of the vertical (*p* < 0.001), horizontal (*p* < 0.05), and transverse planes (*p* < 0.001) of space after the splint therapy. The frequency of the directions of condylar displacements are presented in Table 3.

**Figure 5:**
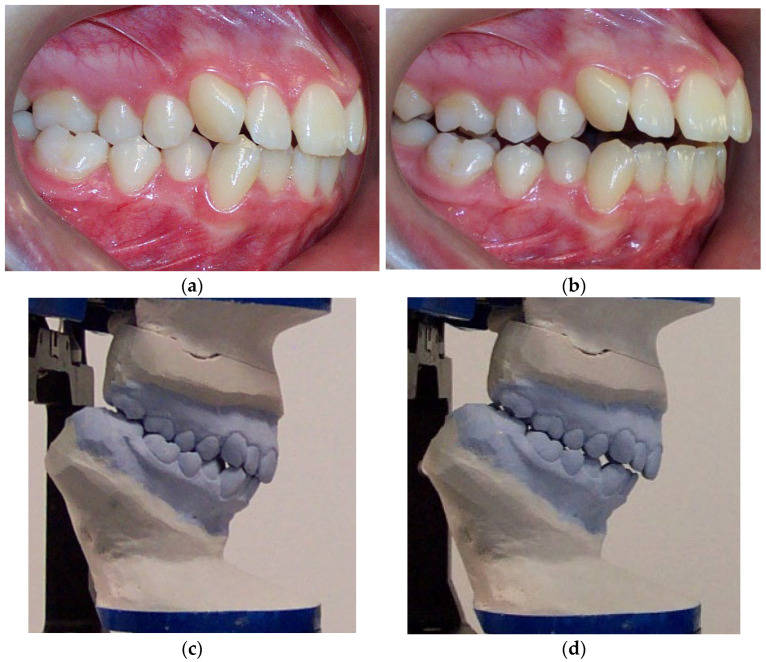
Interarch relationship changes in the same patient, observed intraorally and using stone models mounted on an articulator. (**a**) Pre-splint right lateral intraoral view of occlusion in the MIP. (**b**) Post-splint right lateral intraoral view of occlusion in a stable musculoskeletal position. (**c**) Right lateral view of stone models in the MIP mounted on a semi-adjustable articulator. (**d**) Right lateral view of stone models mounted in the CR after neuromuscular deprogramming with a stabilization splint. The magnitude of the horizontal interarch discrepancy increased while the magnitude of the vertical interarch discrepancy decreased. To accurately evaluate the effect of interarch relationship changes on dentofacial characteristics, a cephalometric analysis of lateral cephalograms was performed before and after the splint therapy (Figure 6).

According to Spearman’s correlation, test results for the anamnestic and diagnostic criteria of RDC/TMD were strongly positively correlated (*p* < 0.05) with the condylar displacements in the vertical and transverse planes before the splint therapy. Interarch relationship changes were observed both intraorally and in models mounted on a semi-adjustable articulator after the orthopedically stable musculoskeletal position was achieved with stabilization splint therapy compared to before the therapy (Figure 5).

**Figure 6:**
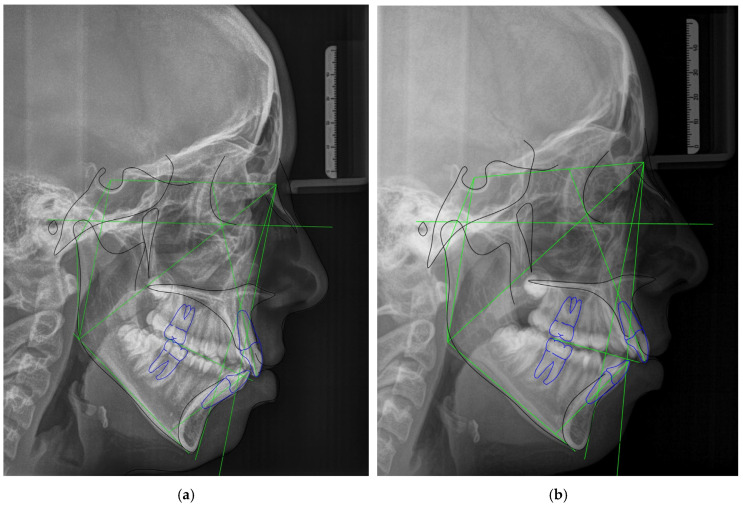
Comparison of pre- and post-splint cephalograms. (**a**) Cephalometric analysis of pre-splint lateral cephalogram. (**b**) Cephalometric analysis of post-splint lateral cephalogram. A comparison of pre- and post-splint cephalograms revealed significantly greater mean values of ANB angle (*p* = 0.007) and Wits appraisal (*p* = 0.022) after splint therapy than those measured before splint therapy. After splint therapy, a significant increase in mean values was observed for overjet (*p* = 0.0001) and a decrease was observed for overbite (*p* = 0.0001) when compared to values obtained before the splint therapy.

There was no significant increase in cephalometric parameters related to the maxillo–mandibular relationship (SNA angle, *p* = 0.431 and SNB angle, *p* = 0.275), vertical skeletal relationship, or the size of the mandible (mandibular length, *p* = 0.958 and ramus height, *p* = 0.985) (Table 4).

When comparing the mean values measured before and after the splint therapy in the female group, we observed a significant increase in the mean values for ANB angle (*p* = 0.006), Wits appraisal (*p* = 0.007), and overjet (*p* = 0.0001), while for overbite, a significant decrease in mean values was found (*p* = 0.0001) after the splint therapy. In the male group, the mean values for overjet significantly increased (*p* = 0.019) and those for overbite significantly decreased (*p* = 0.023) after the splint therapy.

For all other cephalometric parameters related to the maxillo–mandibular relationship, vertical skeletal relationship, and mandible size, a significant difference in the mean values evaluated before and after the splint therapy was not evidenced. It was found that, in patients with a large CR–MI discrepancy after achieving orthopedic stability, the mandible was positioned more posteriorly and rotated in a more clockwise direction, enhancing a hyperdivergent facial type. The result of the Cronbach’s alpha analysis (0.705) was above 0.7, so the sample size was considered reliable.

## 4. Discussion

Earlier investigations indicated orthopedic instability as one of the factors responsible for TMJ and masticatory muscle disorders and pointed to the importance of CR–MI coincidence at the level of the condyles as a guarantee of a stable musculoskeletal position for the condylar processes in the fossa [6,7,8,9,10,11,12,14,15,16,17,21,23,24,25,32,33]. On the other hand, a number of studies reported no association between occlusal factors and TMD, so this issue remains controversial [34,35,36,37]. In addition to the occlusal factors, other etiological factors considered to contribute to the development of TMDs include genetic factors [38], macrotrauma [39], joint hypermobility [40], psychological and psychosocial factors [41,42], parafunctional habits [43], and rheumatic diseases affecting the temporomandibular joint [44]. Regarding orthopedic instability, occlusal interferences are considered to be the most responsible for the development of unstable musculoskeletal position. Increased activity of the lateral pterygoid muscle provoked by a periodontal proprioceptive response from the teeth involved in premature contact causes the mandible to avoid these interferences, displacing the condyles from the fossa [11,45]. Condylar distraction leads to overactivity in the elevator muscles and thus leads to an imbalance between the antagonistic masticatory muscles. The more the condyle is displaced out of the fossa, the more muscle imbalances are enhanced and the discrepancy between the CR and MI increases, imperceptibly leading to the development of TMDs and orofacial pain [45]. To eliminate CR–MI discrepancies and obtain an orthopedically stable position of the condyles in the fossa, the neuromuscular system requires muscle deprogramming, and stabilization splint therapy has been proven to be highly effective for this purpose [9,46]. In our study, articulator mountings of dental casts were performed before and after the orthopedically stable musculoskeletal position was established with stabilization splint therapy, and these were subsequently evaluated for condylar displacements with the CPI device. The results of this study demonstrated that the mean values of condylar displacement obtained after the splint therapy significantly decreased for all three spatial planes in comparison to those measured before the splint therapy. Our findings concurred with the results of similar studies [8,32,47]. All subjects had a significant condylar displacement present in at least one plane of space before splint therapy. Condylar displacements registered before the splint therapy mostly occurred in the posteroinferior, anteroinferior, and straight inferior directions, in agreement with the findings of similar studies [14,15,17,48,49]. After the splint therapy, the direction of the condylar displacement indicated that the condyles were converging toward the CR position as the difference between the CR and MI decreased. This was most distinctly demonstrated by a significant increase in the percentage of minor displacements in the straight inferior and in cases where the CR and MI positions were coincident, with no condylar displacement.

**Figure 7:**
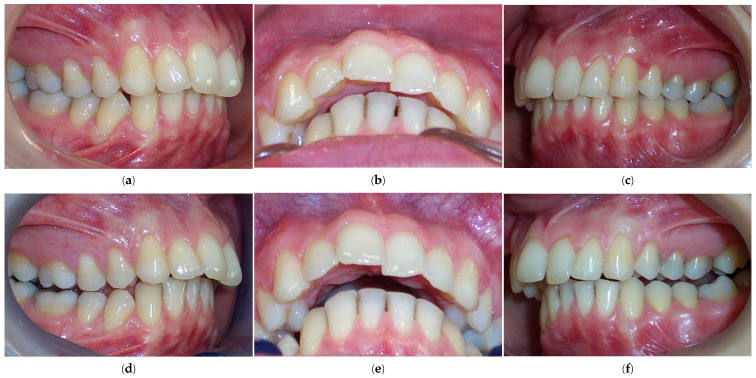
Intraoral view of occlusion in the MI before splint therapy: (**a**) right lateral view; (**b**) inferior overjet view; (**c**) left lateral view. Intraoral view of occlusion in the CR following splint therapy: (**d**) right lateral view; (**e**) inferior overjet view; (**f**) left lateral view. The magnitude of the horizontal (overjet) interarch discrepancy increased and the Class II malocclusion was more pronounced. The magnitude of the vertical (overbite) interarch discrepancy decreased and lower facial height increased.

**Figure 8:**
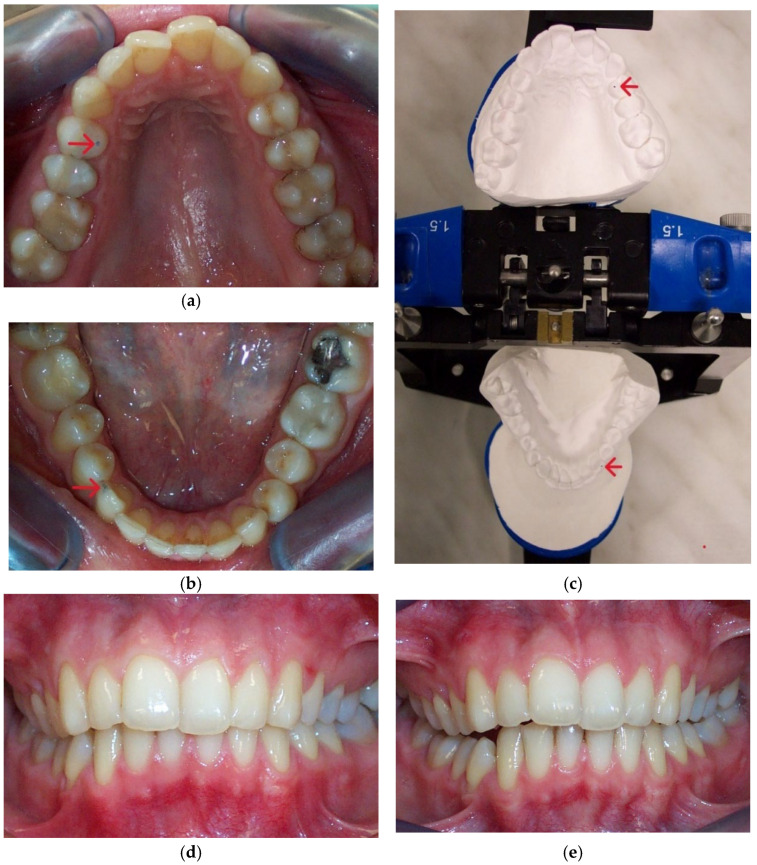
(**a**) Post-splint upper occlusal intraoral view of premature occlusal contact (red arrow); (**b**) post-splint lower occlusal intraoral view of premature occlusal contact. (**c**) Post-splint occlusal view of identic premature occlusal contacts (red arrow) registered in a semi-adjustable articulator. (**d**) Pre-splint frontal intraoral view; (**e**) post-splint frontal intraoral view. A comparison of pre- and post-splint frontal intraoral photographs shows transversely more coincident dental midlines registered after the splint therapy.

The significant reduction in condylar displacement and the achievement of an orthopedically stable musculoskeletal position following the splint therapy directly correlated with the improvement in TMD symptomatology. Hence, the pain related to the area of the temporomandibular joint and masticatory muscles reported by 55% of patients before the splint therapy was completely eliminated after the splint therapy, and this result was the same for intermittent jaw locking. Following the splint therapy, headache symptoms were reduced for more than 90% of patients, corresponding with the study findings of Kemper and Okeson [50]. After the splint therapy, jaw clicking was reduced for more than 80% of patients. Clinical findings regarding the signs of sensitivity to palpation in the masticatory muscles, lateral pole, temporalis tendon, and posterior attachment inside the ear showed a statistically significant reduction in these signs after the splint therapy. Crawford reported a significant increase in the signs and symptoms of TMD as the condylar displacement in vertical and horizontal planes increased from 1 to 2 mm [14]. He et al. demonstrated that 72.9% of pre-treated orthodontic patients with signs and symptoms of TMD had a condylar displacement of greater than 1 mm in the vertical and horizontal planes and 0.5 mm in the transverse plane, while only 11.4% of asymptomatic patients in the control group had increased condylar displacement. They then concluded that the condylar displacements identified in symptomatic patients were a significant contributory factor to the development of signs and symptoms of TMD [16]. This study found that the significant reduction in condylar displacement obtained via splint therapy directly correlated with the improvement in or elimination of signs and symptoms of TMD, concurring with the findings of similar investigations [8,14,23,24,25,32,33]. Moreover, our results agreed with those of authors who found a positive correlation between therapy with an occlusal splint constructed in the CR position and improvements in TMD symptomatology, although in these investigations, a CPI evaluation was not used [51,52,53,54,55]. Furthermore, a significant increase in condylar displacement between the CR and MI generates changes in the dental relationship that can be observed intraorally in the CR position and from dental casts mounted in the CR [15]. The changes in dental interarch characteristics that occur following muscle deprogramming and the establishment of orthopedic stability with stabilization splint therapy are presented in Figure 7 and Figure 8.

The changes in dental interarch relationship characteristics that occur after the stabilization splint therapy might alter the type of dental malocclusion and indirectly affect the orthodontic diagnosis [15,17,21]. To accurately evaluate the impact of interarch relationship changes on facial characteristics and orthodontic diagnostic procedures, an evaluation of the cephalometric data was performed. We analyzed differences in cephalometric measurements between the cephalograms traced in the MIP before, and in the CR position after, the neuromuscular deprogramming and evaluated the eventual changes in dentofacial characteristics. Eight linear and eight angular cephalometric variables were evaluated on pre- and post-lateral cephalograms. Through a comparison before and after the splint therapy, the results of this study showed statistically significant differences for overjet, overbite, ANB angle, and Wits appraisal variables, in agreement with earlier studies in which authors found differences between similar parameters [21,22,47,48]. The significantly larger values of ANB, overjet, and Wits appraisal, and the smaller value of the overbite variable, most substantially contributed to the changes in dentofacial characteristics, whereby most subjects acquired a more dolichofacial skeletal pattern and a retrognathic position of the mandible in the CR position. Our findings were in close agreement with similar investigations by other authors [21,22,23,24,25,47,55]. The values of the angular measurements that determine the vertical skeletal relationship (Bjork polygon angles, articular angle, maxillo–mandibular angle) also increased after neuromuscular deprogramming, but without statistical significance. Nevertheless, an increase in specified angular measurements and a decrease in total anterior/posterior facial height ratio were additional indicators of incurred changes in dentofacial characteristics after the splint therapy (clockwise rotation and posterior positioning of the mandible) was completed. The results of our study were in line with the findings of previous studies, which have shown how substantial changes in dentofacial characteristics that occur after establishing orthopedic stability have a significant effect on orthodontic diagnostic procedures; these studies recommended that an analysis of the lateral cephalograms is carried out from the most stable musculoskeletal position in order to avoid orthodontic misdiagnosis [8,21,22,23,24,25,28,47,48,56,57,58].

Okeson and Ikeda on the basis of their previous findings, in a chapter related to orthodontic therapy and patients with TMD, concluded: “To think that orthodontic therapy could never create risk factors for TMD is a naïve clinical thought. Orthodontists need to establish their treatment goals by considering both the occlusal position and the stable joint position. Establishing orthopedic stability in the masticatory is an important concept for maintaining a healthy masticatory system for a lifetime” [59]. Related to above, and concerning the inconvenience of mounting every patient’s cast, a standardized procedure of mounting the casts of patients with an unstable musculoskeletal position of the condylar processes to identify masked CR–MI discrepancies should be established. Increased hyperdivergence and mandibular retrusion attained as a result of unmasked CR–MI discrepancies could exacerbate Class II malocclusions and cause notable changes in orthodontic treatment planning. These changes imply that the non-extraction case might be converted to an extraction case or non-surgical treatment planning may need to change to surgical treatment planning. Considering the presence of masked occlusal interrelationships in patients with orthopedic instability, the classification of patients according to the type of malocclusion seems irrelevant and unnecessary. The findings of this study suggest that, to identify masked CR–MI discrepancies and avoid orthodontic misdiagnosis, mounting the casts of patients who present with the signs and symptoms of TMD associated with orthopedic joint instability should be a standardized procedure. Furthermore, a substantial amelioration of TMD symptomatology and the achievement of orthopedic condylar stability after splint therapy represent a valid argument for the merits of using the stabilization splint as a standardized procedure for patients with orthopedic instability accompanied by TMDs. In order to reduce the potential risk for the development of signs and symptoms of TMDs, orthodontists should check on orthopedic instability and accordingly provide a means of treatment for this group of patients.

### 4.1. Advantages and Limitations of the Study

The study’s advantage was an analysis of the correlation between TMD symptomatology and condylar displacement in orthodontic patients, addressing the ongoing debate about the relationship between the occlusion and TMD. In order to avoid performance bias, the evaluation of RDC/TMD and CPI and analysis of lateral cephalograms was performed by the same experienced operator.

As a limitation, the study did not include a control group due to ethical concerns related to the diagnostic procedures for orthodontic patients, which made it unjustifiable to require multiple x-rays for those participants. Cone beam computed tomography (CBCT) was used to reconfirm the CPI measurements and observe changes in joint space three-dimensionally. Additionally, the study was conducted at a single center with one operator, which may limit participant diversity and introduce operator bias. Future studies should involve psychologists and sociologists to ensure a thorough assessment of psychosocial distress and pain-related disability among participants.

### 4.2. Further Directions for Research

Longitudinal studies using experimental animal models and MRI should be performed to evaluate the long-term effects of orthopedic instability on the dynamic functions of the masticatory muscle and TMJ system. A greater number of similar studies would provide a sufficient data for conducting a meta-analysis, which would enable orthodontists to thoroughly consider the importance of establishing of an orthopedically stable position for the condyles in the fossa. Further research should be directed towards an investigation of the factors that affect patients’ individual capacity to adapt to an orthopedically unstable musculoskeletal position, as demonstrated by increased CR-MI disharmony. Considering the presence of masked interocclusal relationships in patients with orthopedic instability, the classification of patients according to the type of malocclusion might be excluded from the methodology of future studies. After an orthopedic stability is established by splint therapy, the focus of further research should be on the development of the most convenient treatment strategies to maintain orthopedic stability during the various stages of orthodontic therapy. The effectiveness of occlusal splint therapy performed by an occlusal splint constructed using a conventional articulator and an occlusal splint constructed using a virtual articulator should be compared in future investigations. A greater sample size might further enhance the reliability and validity of the study results.

## 5. Conclusions

The stable musculoskeletal position and more coincident CR–MI relationship obtained after stabilization splint therapy were positively correlated with the reduction in/elimination of the signs and symptoms of TMD.

When the mean condylar displacements registered before and after splint therapy were compared, the displacements after the therapy proved to be significantly lower than those registered before the splint therapy in the case of all three spatial planes; thus, the null hypothesis was rejected.

During the change from an orthopedically unstable (MI) to an orthopedically stable musculoskeletal position (CR), the mandible moved backward and rotated clockwise, contributing to a more dolichofacial skeletal pattern.

The orthopedic stability provided by stabilization splint therapy opens up the opportunity for more accurate orthodontic diagnoses using the orthopedically stable musculoskeletal position. When indicated, orthodontists are required to establish a musculoskeletally stable relationship between the positions of MI and CR in order to prevent orthodontic misdiagnosis, ameliorate TMDs, and reduce potential risk factors for the development of signs and symptoms of TMD. There were no statistically significant sex differences.

## Figures and Tables

**Table 1:** Comparison of the mean ± standard deviation values of self-reported facial pain evaluated before and after orthopedic stability was achieved with a stabilization splint.

The Level of Facial Pain’s Interference with Daily Activities, Rated on a 0–10 NRS				
n = 33	Mean		Z	*p* Value
	Before	After		
How would you rate your facial pain on a 0 to 10 scale at the present time, which is right now, where 0 is “no pain” and 10 is “pain as bad as could be”?	5.88 ± 2.25	1.67 ± 0.58	−2.868	0.004
In the past six months, how intense was your worst pain rated on a 0 to 10 scale, where 0 is “no pain” and 10 is “pain as bad as could be”?	7.97 ± 1.63	2.33 ± 0.58	−2.892	0.004
In the past six months, on average, how intense was your pain rated on a 0 to 10 scale, where 0 is “no pain” and 10 is “pain as bad as could be”? [That is, at times you were experiencing your usual pain].	6.88 ± 1.65	2.00 ± 0.00	−2.887	0.004
In the past six months, how much has facial pain interfered with your daily activities rated on a 0 to 10 scale, where 0 is “no interference” and 10 is “unable to carry on any activities”?	5.64 ± 2.22	1.33 ± 0.58	−2.716	0.007
In the past six months, how much has facial pain changed your ability to take part in recreational, social, and family activities, where 0 is “no interference” and 10 is “extreme change”?	5.79 ± 2.33	1.00 ± 0.00	−2.777	0.005
In the past six months, how much has facial pain changed your ability to work (including housework), where 0 is “no interference” and 10 is “extreme change”?	5.42 ± 2.19	1.00 ± 0.00	−2.773	0.006

**Table 2:** Comparison of jaw dysfunction symptoms of TMD evaluated before and after the splint therapy.

Symptoms of TMD Related to Jaw Dysfunction				
n = 60	Before the Splint Therapy		After the Splint Therapy	
		Percentage		Percentage
Have you ever had your jaw lock or catch so that it won’t open all the way?	24	40%	0	0.0%
Does your jaw click or pop when you open or close your mouth or when chewing?	42	70.0%	5	8.3%
Does your jaw make a grating or grinding noise when it opens and closes or when chewing?	6	10.0%	1	1.7%
Have you been told or do you notice that you grind your teeth or clench your jaw while sleeping at night?	5	8.3%	1	1.7%
During the day, do you grind your teeth or clench your jaw?	3	5.0%	0	0.0%
Does your jaw ache or feel stiff when you wake up in the morning?	10	16.7%	0	0.0%
Do you have noises or ringing in your ears?	2	3.3%	0	0.0%
Does your bite feel uncomfortable or unusual?	42	70.0	0	0.0%

χ^2^ = 27.514; *p* = 0.0001.

**Table 3:** Comparison of changes in condylar position evaluated before and after the splint therapy.

Changes in the Direction of Condylar Displacement				
n = 60	Before the Splint Therapy		After the Splint Therapy	
	Right	Left	Right	Left
	Percentage		Percentage	
Posterior–inferior	66.7%	75%	41.6%	48.3%
Anterior–inferior	26.6%	20%	6.7%	11.7%
Straight inferior	6.7%	5%	46.7%	33.3%
Coincident CR-MI	-	-	5%	6.7%

**Table 4:** Comparison of mean ± standard deviation values of cephalometric variables analyzed before and after splint therapy.

n = 60	Before Splint Therapy Mean	After Splint Therapy Mean	Z	*p* Value
**Maxillo–mandibular relationship**				
SNA angle (°)	81.30 ± 4.01	81.84 ± 3.98	−0.787	0.431
SNB angle (°)	78.86 ± 4.58	78.08 ± 4.89	−1.092	0.275
ANB angle (°)	2.43 ± 2.54	3.73 ± 2.80	−2.704	0.007
Wits appraisal (mm)	0.59 ± 3.15	1.57 ± 3.51	2.286	0.022
**Vertical skeletal relationship**				
Saddle angle (N-S-Ar) °	120.61 ± 5.6	120.23 ± 5.67	−0.299	0.765
Articular angle (S-Ar-Go) °	146.68 ± 7.75	148.67 ± 7.07	−1.441	0.150
Mandibular angle (Ar-Go-Gn) °	127.28 ± 7.99	127.67 ± 7.92	−0.381	0.704
Sum of angles °	394.60 ± 7.43	395.61 ± 7.44	−1.559	0.119
Total anterior facial height (mm)	115.66 ± 6.73	117.27 ± 7.03	−1.176	0.24
Total posterior facial height (mm)	75.49 ± 7.65	75.31 ± 7.48	−0.060	0.952
Total anterior/posterior facial height	65.28 ± 5.55	64.22 ± 5.33	−0.963	0.335
(S-Go/N-Me) %				
Maxillo–mandibular angle (PP-MP) (°)	28.79 ± 7.47	29.84 ± 7.38	−0.672	0.502
**Size of mandible**				
Mandibular length (Go-Me) mm	69.21 ± 5.72	69.18 ± 5.53	−0.052	0.958
Ramus height (Ar-Me) mm	43.81 ± 5.92	43.74 ± 5.97	−0.018	0.985
**Dental relationship**				
Overjet (mm)	3.14 ± 1.76	4.79 ± 2.32	4.856	0.0001
Overbite (mm)	1.75 ± 1.80	0.45 ± 1.59	−4.119	0.0001

## Data Availability

Data are contained within the article.
